# Detection of novel *Plasmodium falciparum* coronin gene mutations in a recrudescent ACT-treated patient in South-Western Nigeria

**DOI:** 10.3389/fcimb.2024.1366563

**Published:** 2024-04-23

**Authors:** Olusola Ajibaye, Yetunde Adeola Olukosi, Eniyou C. Oriero, Mary Aigbiremo Oboh, Bamidele Iwalokun, Ikechukwu Chidiebere Nwankwo, Chinaza Favour Nnam, Olawunmi Victoria Adaramoye, Somadina Chukwemeka, Judith Okanazu, Eniafe Gabriel, Emmanuel Oluwadare Balogun, Alfred Amambua-Ngwa

**Affiliations:** ^1^ Malaria Genomics Research and Training Centre, Department of Biochemistry & Nutrition, Nigerian Institute of Medical Research, Yaba, Lagos, Nigeria; ^2^ Medical Research Council Unit, the Gambia – The London School of Hygiene and Tropical Medicine, Fajara, Banjul, Gambia; ^3^ Center for Molecular Parasitology, Department of Microbiology and Immunology, Drexel University College of Medicine, Philadelphia, PA, United States; ^4^ Department of Obstetrics and Gynaecology, Lagos University Teaching Hospital, Idi-araba, Surulere, Lagos, Nigeria; ^5^ Department of Biochemistry, Ahmadu Bello University, Zaria, Nigeria; ^6^ Department of Biomedical Chemistry, Graduate School of Medicine, The University of Tokyo, 7-3-1 Hongo, Bunkyo-ku, Tokyo, Japan; ^7^ Center for Discovery and Innovation in Parasitic Diseases, Skaggs School of Pharmacy and Pharmaceutical Sciences, University of California San Diego, Gilman Drive, La Jolla, CA, United States

**Keywords:** malaria, Plasmodium falciparum, coronin, polymorphisms, artemisinin resistance, lumefantrine

## Abstract

**Background:**

Routine surveillance for antimalarial drug resistance is critical to sustaining the efficacy of artemisinin-based Combination Therapies (ACTs). *Plasmodium falciparum kelch-13 (Pfkelch-13)* and non-*Pfkelch-13* artemisinin *(*ART) resistance-associated mutations are uncommon in Africa. We investigated polymorphisms in *Plasmodium falciparum* actin-binding protein (Pfcoronin*)* associated with *in vivo* reduced sensitivity to ART in Nigeria.

**Methods:**

Fifty-two *P. falciparum* malaria subjects who met the inclusion criteria were followed up in a 28-day therapeutic efficacy study of artemether-lumefantrine in Lagos, Nigeria. Parasite detection was done by microscopy and molecular diagnostic approaches involving PCR amplification of genes for *Pf*18S rRNA, *var*ATS, telomere-associated repetitive elements-2 (TARE-2). *Pfcoronin* and *Pfkelch-13* genes were sequenced bi-directionally while clonality of infections was determined using 12 neutral *P. falciparum* microsatellite *loci* and *msp2* analyses. Antimalarial drugs (sulfadoxine-pyrimethamine, amodiaquine, chloroquine and some quinolones) resistance variants (DHFR_51, DHFR_59, DHFR_108, DHFR_164, MDR1_86, MDR1_184, DHPS_581 and DHPS_613) were genotyped by high-resolution melting (HRM) analysis.

**Results:**

A total of 7 (26.92%) cases were identified either as early treatment failure, late parasitological failure or late clinical failure. Of the four post-treatment infections identified as recrudescence by *msp2* genotypes, only one was classified as recrudescence by multilocus microsatellites genotyping. Microsatellite analysis revealed no significant difference in the mean allelic diversity, *He*, (P = 0.19, Mann-Whitney test). Allele sizes and frequency per locus implicated one isolate. Genetic analysis of this isolate identified two new *Pfcoronin* SNVs (I68G and L173F) in addition to the P76S earlier reported. Linkage-Disequilibrium as a standardized association index, *I_A_^S^*, between multiple *P. falciparum* loci revealed significant LD (*I_A_^S^* = 0.2865, *P*=0.02, Monte-Carlo simulation) around the neutral microsatellite loci. The *pfdhfr/pfdhps/pfmdr1* drug resistance-associated haplotypes combinations, (108**_T/N_**/51**_I/_** 164**_L_**/59**_R_**/581**_G_**/86**_Y_**/184**_F_**), were observed in two samples.

**Conclusion:**

*Pfcoronin* mutations identified in this study, with potential to impact parasite clearance, may guide investigations on emerging ART tolerance in Nigeria, and West African endemic countries.

## Introduction

The emergence and spread of resistance to antimalarial drugs in *Plasmodium* spp. and resistance to insecticides in the mosquito vectors are the two leading limitations to the control of malaria (WHO, 2023). In sub-Sahara Africa (sSA), the first-line treatment for the most common malaria parasite, *Plasmodium falciparum*, is artemisinin-based combination therapies (ACTs), which include artemether-lumefantrine (AL), artesunate-amodiaquine (AS-AQ) and dihydroartemisinin-piperaquine (DHA-PPQ) (Yobi et al., 2020). Cases of clinical failure of these combination drugs or their components have been reported in Africa, sparking fears of reduced efficacy or outright resistance. In the historical paths to resistance, failure of ACTs in Asia has been linked to mutations in the *P. falciparum* genome, with variants in the Kelch13 gene (*Pfk13*) associated with resistance to artemisinin derivatives (White, 2004; Taylor et al., 2018; Conrad and Rosenthal, 2019; WWARN, 2019; Ajibaye et al., 2020; Ikegbunam et al., 2021). Meanwhile, resistance to partner drugs is well known and associated with mutations in several genes, including, chloroquine resistance transporter (*Pfcrt* codon 72 to 76, haplotype SVMNT), multidrug resistance protein-1(*Pfmdr1:* N86F, N86Y, Y184F, S1034C) for amodiaquine resistance, and multiple copies of plasmepsin genes (*Pfpm2*) for piperaquine resistance (Yobi et al., 2020). Beyond these, single nucleotide variants in the *P. falciparum* actin-binding protein- Pfcoronin (G50E, R100K, and E107V) and *Pfap2-mu* have been implicated in increased *in vitro* tolerance to artemisinin, linked to kelch-13-independent mechanisms (Demas et al., 2018; Henrici et al., 2019; Stokes et al., 2021). The frequency of artemisinin resistance-associated variants in natural malaria parasite infections in Africa remains low and their roles in clinical response to ACTs remain unclear. As other *P. falciparum* genetic factors may be mediating *kelch13*-independent artemisinin tolerance mechanisms in Africa, improved continuous genetic surveillance and functional studies will be useful in effectively assessing and strategizing treatment regimens to prolong the effectiveness of current malaria treatments. Additionally, several non-Asian type *Pfkelch-13* variants associated with decreased sensitivity to artemisinin are reported across Africa, though in low frequencies (Stokes et al., 2021). With continuous use of ACTs for first-line treatment and consideration for its use for chemopreventive mass interventions, these common African variants of *Pfkelch-13* could be selected to become dominant as observed in Rwanda and Uganda (Sharma et al., 2020). Their presence and spread across Africa will severely impede the efficacy of artemisinin-based treatments. Currently, there is only one report for Pfcoronin involvement in antimalarial drug resistance, but this report is an *in vitro* study (Demas et al., 2018; Henrici et al., 2019). Hence, whether *Pfcoronin* SNPs may be associated with parasite ART tolerance *in vivo* is not known.

Further research and investigation are necessary to ascertain the relationship between Pfcoronin mutations and reduced ART susceptibility in clinical setup.

Pfcoronin is a WD40-propeller domain protein family member that shares the β-propeller motif with *Pf*Kelch13 protein (Adams et al., 2000). In eukaryotes, Kelch and Coronin proteins have distinct functions, some Kelch domain-containing proteins are also actin-binding proteins, a function typical of Coronin proteins. Only one single-copy of *coronin* gene is encoded by the genomes of unicellular pathogens, including *P. falciparum* (Xavier et al., 2008). The encoded protein generally binds F-actin and plays significant roles in proliferation, locomotion, and phagocytosis in eukaryotes (Xavier et al., 2008).

In addition to routine *in vivo* monitoring of antimalarial efficacy, the WHO recently released a strategy for genomic surveillance of infectious pathogens, emphasizing its contribution to disease surveillance and intervention policy (WHO, 2022). For the major malaria parasite, *P. falciparum*, several molecular surveillance approaches for drug resistance analysis based on microsatellites and SNP barcodes as well as highly diverse surface antigens have been in existence. The Genomes of several thousand isolates are now also publicly available, allowing for mining more markers, including those related to resistance to previous and current drugs. These combined, allow for temporal and spatial monitoring of the effect of interventions on diversity and the evolution of important phenotypes such as drug and vaccine resistance. The increasing pressure from interventions for malaria elimination in high transmission areas such as Nigeria, and consequent discontinuity in spatial flow of infections as populations dwindle may result in sub-structuring with selection and establishment of emerging drug resistance in response to sustained drug use, and reduced recombination (Auburn et al., 2011).

Several molecular diagnostic techniques have been validated, the Taqman *var* acidic terminal sequence (*varATS*) -based real-time PCR method is ultra-sensitive, very reliable, but relatively costly. Especially for resource limited settings. Hence, the need for cheaper, acceptably sensitive methods adaptable in such settings. Malaria transmission in Nigeria is substantially heterogeneous with seasonality observed in the North and perennial transmission in the South (Snounou and Beck, 2002; Selvaraj et al., 2018; Oladipo et al., 2022). Endemicity shows graduation throughout the country. Despite the considerable increase in malaria control efforts by the National Malaria Elimination Programme (NMEP), Nigeria remains one of the countries with the highest incidence of malaria morbidity and mortality (WHO, 2023). Various ACTs are easy to access for treatment across the country, while Sulphadoxine-Pyrimethamine and Amodiaquine are used for seasonal malaria chemoprevention in the northern Sahel regions. This may potentiate emergence of drug tolerance by the circulating drug resistant *P. falciparum* strains in the country. This study surveyed isolates from a therapeutic efficacy study in Lagos (Southwest Nigeria) for single nucleotide polymorphisms (SNPs) in *P. falciparum* antimalarial resistance markers, including *Pfcoronin*, which is associated with reduced *in vitro* sensitivity to artemisinin (Demas et al., 2018). The genetic diversity of this *P. falciparum* population was also evaluated.

## Methods

### Study design and ethical considerations

This antimalarial drug surveillance study was a one-arm prospective evaluation of clinical and parasitological responses to observed treatment for uncomplicated malaria following the guidelines of the WHO Global Malaria Programme updated in 2021 (http://www.who.int/malaria/areas/drug_resistance/en/).

Participants with uncomplicated malaria who met the study inclusion criteria were enrolled, treated on site with Artemether-lumefantrine (AL) and monitored for 28 days. The follow-up consisted of a fixed schedule of check-up visits and corresponding clinical and parasitological examinations. On the basis of the results of these assessments, the patients were classified as having therapeutic failure (early or late) or an adequate response. The proportion of patients experiencing therapeutic failure during the follow-up period was used to describe AL efficacy in the population. Molecular analysis was used to distinguish between recrudescence and episodes of reinfection.

**Figure 1:**
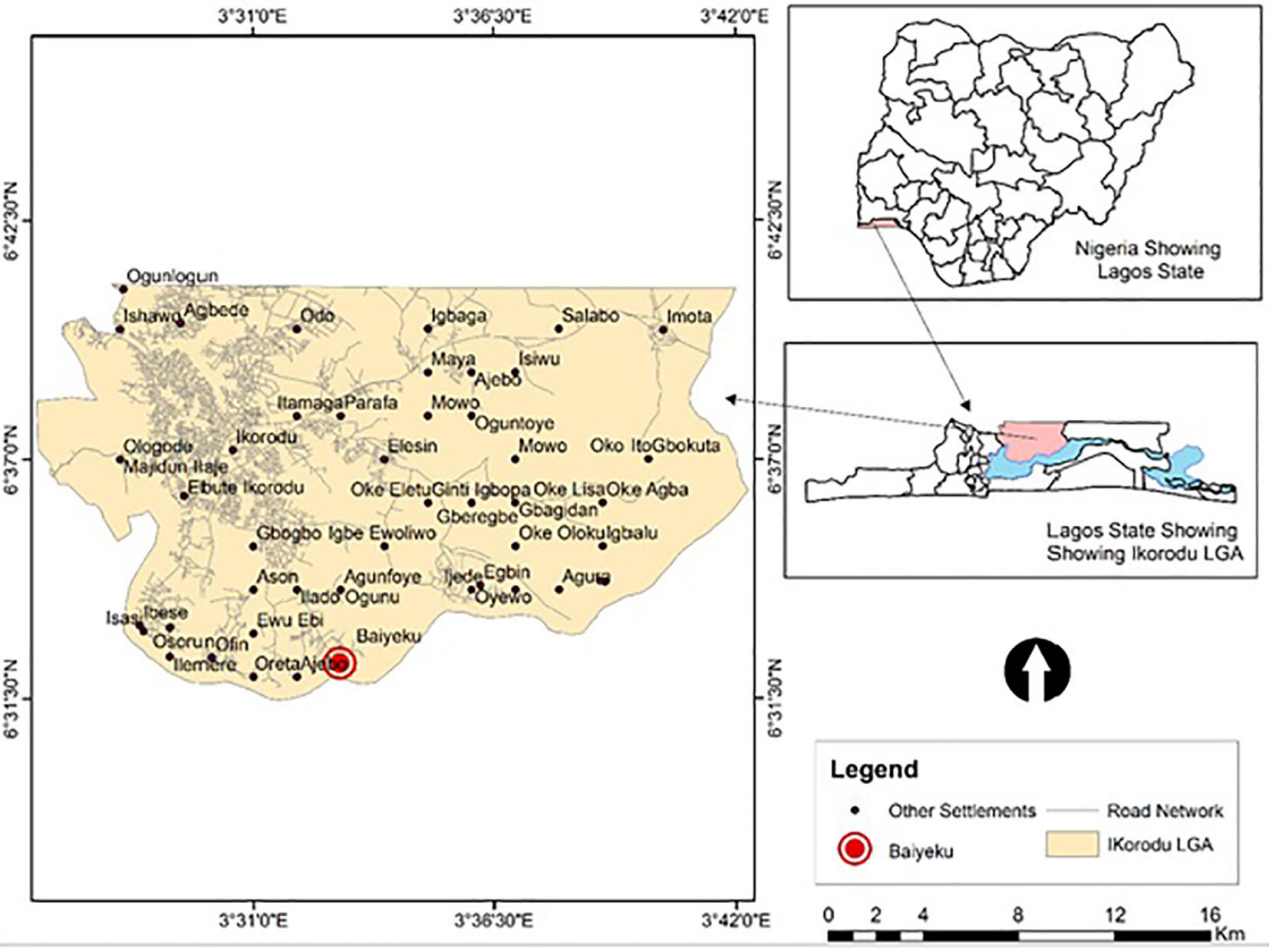
Map of study site. Top Right: Map of Nigeria showing the location of Lagos State; Down Right: Zoomed-Map of Lagos showing Location of Ijede; Left: Map of Ijede showing the location of Ijede General Hospital (IJGH). Pink: Study area including the Health facility; Light blue: Areas bordering water bodies around the study site.

This study was carried out at Ijede General hospital (IJGH), Ijede, Ikorodu, Lagos, South-West, Nigeria. Ijede is a rural settlement, located along the Lagos Lagoon on Latitude 6^°^ 34’ 00’’N and Longitude 3^°^ 35’ 18’’ (**Figure 1**); bordering Ogun State. The community has an estimated population of 535,619 inhabitants.

Only participants who consented were included in the study after they or parents or guardians of those who were children gave informed consent. Ethical approval for the study was obtained from the Institutional Ethics Review Board of the Nigerian Institute of Medical Research (NIMR IRB No: IRB/21/030). All the research participants gave written informed consent, guardian/parental consent and/or assent (for participants aged 7- 11 years).

Participants in this study were primarily recruited for *in vivo* therapeutic efficacy study (TES) of Artemether-Lumefantrine (AL) conducted between July and September 2019. Inclusion criteria included, (1) being aged ≥ 12 months to <70 years (for ease of follow-up), (2) symptoms suggestive of uncomplicated malaria including an axillary temperature of ≥ 37.5 °C or a history of fever in the last 24 hours preceding presentation, (3) confirmed microscopically, *P. falciparum* mono-infection (Day 0) with asexual parasite counts ≥ 2,000/μl. Individuals presenting at this facility were initially screened with histidine-rich protein 2-based malaria rapid diagnostic test kits (HRP2-mRDTs). All test results were simultaneously confirmed by microscopy and later on with molecular methods at all time-points. Giemsa-stained thick blood films were read with 200 white blood cells (WBCs) counted by light microscopy. Parasite density (asexual parasites per microlitre of blood), was calculated as number of asexual parasites divided by the number of WBCs counted (200) and then multiplying it by an assumed WBC number (8000 cells/L). Individuals with severe malaria, malnutrition, serious underlying diseases (renal, cardiac, or hepatic diseases), severe malnutrition, severe danger signs, or signs of severe malaria, and known allergies to AL were excluded from the study. Individuals with other relatively common febrile conditions like otitis media, tonsillitis, measles and abscesses were also excluded. Pregnant (as determined by the human chorionic gonadotropin test) and lactating women were excluded. Participants received AL (Coartem) (Novartis, Switzerland) according to the Nigeria Federal Ministry of Health (FMoH) treatment guideline and directly observed closely. For adults (at least 35 kg), 4 tablets taken orally as a single initial dose, followed by 4 tablets after 8 hours, and then 4 tablets twice a day (morning and evening) for the following 2 days, making a total course of 24 tablets. For children (5 to less than 15 kg), 1 tablet taken orally as a single initial dose, followed by 1 tablet after 8 hours, and then 1 tablet twice a day (morning and evening) for the following 2 days (total course of 6 tablets); Weight 15 to less than 25 kg, 2 tablets taken orally as a single initial dose, followed by 2 tablets after 8 hours, and then 2 tablets twice a day (morning and evening) for the following 2 days, a total course of 12 tablets; Weight 25 to less than 35 kg, 3 tablets orally as a single initial dose, followed by 3 tablets after 8 hours, and then 3 tablets twice a day (morning and evening) for the following 2 days for a total course of 18 tablets.

Participants were followed up on days 1 to 3 and then on days 7, 14, 21 and 28 for parasite clearance. Antimalarial drugs were administered as directly observed therapy (DOT), after normal meal, while the participants visited the health facility. Participants were treated with Dihydroartemisinin-piperaquine in cases where infection did not resolve after AL administration. Early Treatment Failure (ETF) was taken as persistence of parasitaemia up till day 3 with axillary temperature ≥ 37.5°C; Late Parasitological Failure (LPF) was parasitaemia on any day between day 7 and day 28 with axillary temperature ≥ 37.5°C; Late Clinical Failure (LCF) was presence of danger signs or severe malaria or parasitaemia with axillary temperature ≥37.5°C between day 4 and day 28, without having been previously classified as ETF while Adequate Clinical and Parasitological Response (ACPR) was taken as absence of parasitaemia on day 28, in patients who did not previously meet any of the criteria for ETF or LTF. Two WHO certified microscopists read the slides, and the third read any slide with differing microscopy readings to resolve discrepancies. A 3 mL venous blood sample was collected into ethylene diamine tetra-acetic acid (EDTA) coated vacutainer bottles (Becton Dickinson) at commencement of treatment and each time point during follow up. To prepare parasite isolates for genotyping, 2 mL of each sample was depleted of human leukocytes by filtering through cellulose columns (Tiwari et al., 2012; Venkatesan et al., 2012; Rutledge et al., 2017). Each blood sample was also spotted on Whatman 3 filter paper (Whatman International, England) for genomic DNA (gDNA) extraction and other molecular assays.

#### Molecular diagnosis of infection with malaria parasites

Genomic DNA from each sample was extracted with the Qiagen blood Mini kit (CAT 51306, Qiagen, Hilden, Germany), and quantified with NanoDrop One Instrument (Thermo Fisher Scientific™, Wilmington, USA) (Desjardins and Conklin, 2010). Following microscopic examination, infection with *P. falciparum* for each sample was confirmed with three described molecular diagnostic techniques targeting the 18S srRNA gene in conventional and real-time PCR; the highly sensitive variable Acidic terminal sequence (*var*ATS) and Telomere Associated Repetitive sequence (TARE)-2 by real-time PCR (Hofmann et al., 2015). For real time assays, samples were negative when cycle time (Ct) was ≥ 40. For the coupled PCR, the 18S primary PCR product was used as template for the real-Time PCR (Rutledge et al., 2017). Primers were synthesized by Macrogen Inc, Seoul, Korea.

#### Molecular genotyping of primary and recurrent infections

Each sample classified as positive for *P. falciparum* was first genotyped by amplifying the gene of merozoite surface protein II (*msp2*). The *msp2* fragment sizes were used to classify new infections from recrudescence in the pre- and post-treatment parasite isolates. Genotyping *msp2* employs nested-PCR to amplify the block 3 of *msp2* containing two allelic families (Snounou, 2002; Snounou and Beck, 2002; Hofmann et al., 2015). Band size variability of less than 18 bp was taken as identical *msp2* alleles. To further determine *P. falciparum* genetic diversity and pairwise similarity between infections from different time-points and individuals, 12 unlinked multi-locus microsatellite markers were amplified using a heminested PCR as described previously (Anderson et al., 2006; Druml et al., 2006; Murray et al., 2016). The loci analyzed included Polyα (Chr4), TA81(Chr5), PfPk2 (Chr12), ARAII (Chr11), TA87 (Chr 6), PfG377 (Chr12), TA40 (Chr10), TA60 (Chr13) and TA109 (Chr 6). The final labeled (FAM, HEX or PET dyes) heminested PCR products were first visualized on 1.5% agarose gel and further analyzed by capillary electrophoresis on a SeqStudio™ Genetic Analyzer (Applied Biosystems™, Foster City, CA, USA) together with GeneScan™ 600 LIZ internal size standards (Applied Biosystems, Foster City, CA) and for normalization across runs. Allele length and peak height per locus and samples were scored using GeneMapper software (Thermo Fisher Scientific, Dartford, UK). Minor alleles were scored when the minor peaks were ≥ 25% the height of the predominant allele in the isolate with a relative fluorescence unit of at least 100. Fragments within ±7bp sizes were binned. Multiple infections were defined when any of the loci contained multiple alleles at any of the microsatellite loci genotyped. Multilocus genotype similarity was inferred for pairs of isolates with the same allele sizes across all loci.

### Drug resistance marker genotyping

We employed the high-resolution melting (HRM) Assay panel which simultaneously detects SNPs in three *P. falciparum* genes *(pfmdr, pfdhps and pfdhfr)* that are associated with antimalarial drug resistance (Daniels et al., 2012). PCR reactions were carried out using the Type-it HRM PCR kit (Qiagen, Germany) according to manufacturers’ protocol. Briefly, the reaction contained 1× HRM PCR master mix, 0.7µM (1×) of primers (S2) from a 10× primer mix and 1 µL DNA (~1 ng/µL) in a total reaction volume of 10 µL. Amplification cycle was 95°C denaturation for 5 min, followed by 50 cycles of 95°C for 10 s and 55°C for 30 s and 72°C for 10 s with single acquisition, and then a pre-melt cycle of 1s at 65°C followed by continuous fluorescence data acquisition (10 acquisitions per second) at 95°C. Products were then cooled to 40°C till end of the amplification cycle.

#### Plasmodium falciparum Kelch-13 (Pfk13) and coronin (pfcoronin) sequencing

An 848 bp *Pfkelch-13* gene fragment was amplified from each infected sample by nested-PCR using the following primer sets: pfk13-F: 5′-CGGAGTGACCAAATCTGGGA-3′ and pfk13-R: 5′-GGGAATCTGGTGGTAACAGC-3′ for the primary reactions and pfk13-F: 5′-GCCAAGCTGCCATTCATTTG-3′ and pfk13-R: 5′-GCCTTGTTGAAAGAAGCAGA-3′ for the nested reaction. *Pfkelch-13* PCR cycling conditions for both primary and nested reactions included 5 minutes of initial denaturation at 95°C followed by 40 cycles of 30 s denaturation at 94°C, 90 s annealing at 60°C, 90 s extension at 72°C and a 10 minutes final extension at 72°C. All PCR reactions were carried out with 2 μL of DNA in a total volume of 25 μL containing 0.2 mM dNTPs, 2 mM MgCl_2_, 1 μM of each primer, and 1 unit of PfuUltra II Fusion HS DNA polymerase (Agilent Technologies, Santa Clara, USA). For the *pfcoronin*, a 542 bp fragment was amplified with the following primers pfcoro-F: 5’-ATGGCAAGTTGAAGGGGGAG-3’ and pfcoro-F:5’-TTGTCTTCACCACCAAATCCA-3’ in a one-step PCR amplification. Cycling conditions for the amplification of the *pfcoronin* were 95°C initial denaturation for 5 min, 40 cycles of denaturation at 95°C for 30 s, annealing at 56°C for 45 s and extension at 68°C for 30 s, these were followed by final extension at 72°C for 7 min. All amplicons were resolved on 1% agarose gel with ethidium bromide staining. PCR amplicons for sequencing were purified with Genomic DNA Clean & Concentrator-10 DNA clean up kit (Zymo Research, Irvine, USA). The purified DNA was sequenced bi-directionally on SeqStudio automatic DNA sequencer (Applied Biosystems™, Foster City, USA) using the BigDye Terminator Cycle Sequencing Kits (Applied Biosystems™, Foster City, USA) according to manufacturer’s instructions with the PCR amplification primers. Samples were re-sequenced to confirm SNVs.

### Statistical and population genetic analyses

Parametric demographic data generated from the study were analyzed using One-way ANOVA and Tukey for multiple comparison while non-parametric microsatellite data were analyzed using Kruskal-Wallis and Dunn’s multiple comparison and Mann-Whitney test. Test of statistical significance was set at < 0.05 and confidence interval at 95%.

The allele frequencies and number of alleles per locus were calculated using GENALEX 6 [26]. Allelic diversity for each of the microsatellite loci was calculated using the formula for expected heterozygosity *H_E_* = [(n/n-1) (1-∑p^2^)], where n is the number of isolates analyzed and p represents the frequency of each different allele at a locus. *H_E_* is the proportion of heterozygosity expected under random mating and pi is the allele frequency of the i-th allele. Differences between the allelic diversity (*H_E_* of the two groups *i.e.* pre and post drug treatment groups) was assessed using Mann-Whitney test in GraphPad Prism 8 (GraphPad Software, Inc). *H_E_* has a potential range from 0 (no allele diversity) to 1 (all sampled alleles are different).

Linkage Disequilibrium microsatellite loci expressed as the standardized index of association (*I_A_^S^*), was determined using the web-based interface, LIAN version 3.7 (Haubold and Hudson, 2000; Peakall and Smouse, 2006). *I^S^_A_* = (1/*n* – 1 ((*V_D_*/(*V_E_*) – 1), where *n* is the number of loci for which two individuals differ, *V_E_* and *V_D_* are the expected and observed variance, respectively. Values range from 0 (no loci in LD) to 1 (all loci in LD). The null hypothesis of complete linkage equilibrium (*I_A_^S^* = 0) was tested by Monte-Carlo simulations using 10,000 random permutations of the data (Kumar et al., 2016). This genetic phenomenon follows recurrent recombination between alleles at distinct loci in LIAN to compare the variance in the number of alleles shared between all pairs of genotypes with the variance expected under a random association hypothesis. We assessed the genetic relatedness between pre- and post-drug infection haplotypes by calculating pairwise allele sharing (P_AS_). Specifically, P_AS_ was calculated as the number of alleles shared between two infection (pre- and post-) haplotypes divided by the number of microsatellite markers (i.e., 12). Multivariate analysis of similar alleles to determine genetic variance among isolates Pre- and Post-AL administration was done by Principal Component Analysis (PCA) using the libraries Tidy-verse, Broom, Cowplot and ggplot2 in R (http://CRAN.R-project.org/) based on microsatellite data.

For *Pfkelch-13* and *Pfcoronin* sequence analysis, chromatograms were manually edited and aligned to create sample contigs using Bioedit software. Contigs were then aligned with CLUSTAL W in MEGA 6 (Haubold and Hudson, 2000; Anderson et al., 2006; Peakall and Smouse, 2006). Multiple sequence alignments were compared against reference sequences of *Pfkelch-13* (PF3D7_1251200) and *Pfcoronin* 3D7 (PF3D7_125200) strains and plasmodium *spp* homologues retrieved from the GenBank. SNPs were called using Geneious Prime software 2021.1.1 and SNPServer (http://hornbill.cspp.latrobe.edu.au/snpdiscovery.html). The number of haplotypes were analyzed using DnaSP version 6.12.03. The distance between sequences was assessed using a neighbor-joining (NJ) tree from Kimura-2-parameter distance matrix in MEGA 6 (Saitou and Nei, 1987; Tamura et al., 2004; Kumar et al., 2016) The consensus distance tree was displayed and visualized as a dendrogram.

### Structure-function effect analysis of SNPs

Delta Delta G (DDG), a metric for predicting how a single point mutation will affect protein stability, often referred to as ΔΔG, is the change in Gibbs free energy. To predict the effects of non-synonymous SNPs on the structure and function of the identified *P. falciparum* coronin, a web-based tool, PremPS (Chen et al., 2020), was used with conservation score from alignment of threading template.

The robustness was confirmed with Polyphen-2 (http://genetics.bwh.harvard.edu/pph2/). The 3D structure of the wild-type protein was obtained from the Protein data bank (https://swissmodel.expasy.org/interactive/4avQwh/templates/). The structures of mutants were produced with Build Model module of FoldX (http://foldxsuite.crg.eu/) using wild-type protein structure as the templates. We analyzed functional and structural stability using the change in Gibbs free energy expressed as. Delta Delta G (ΔΔG) between wild type and mutant PfCoronin protein. ΔΔG measures the change in energy between the folded and unfolded states (ΔGfolding) and the change in ΔGfolding when a point mutation is present.

## Results

**Figure 2:**
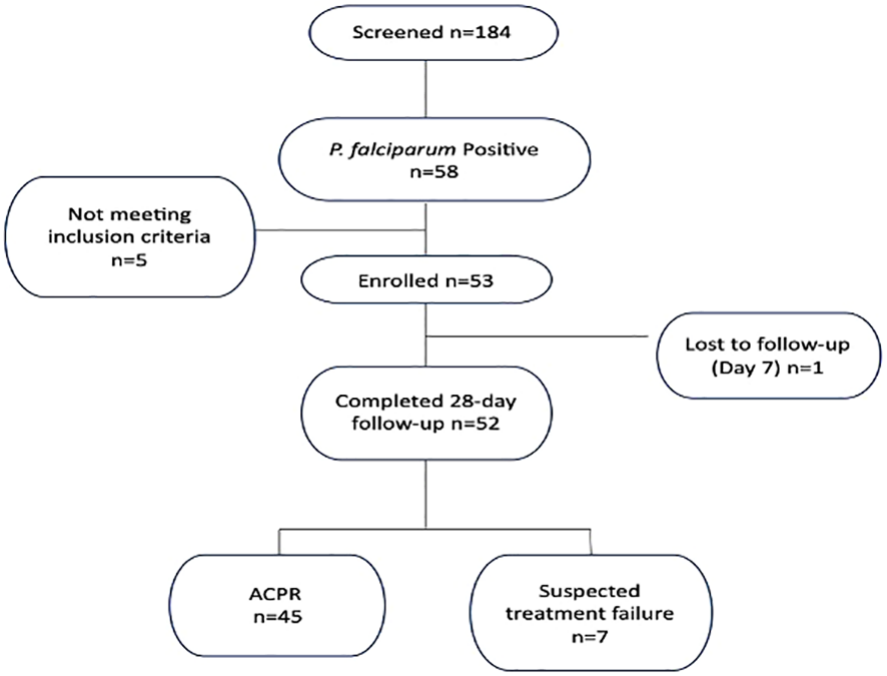
Enrollment and treatment outcomes.

**Figure 3:**
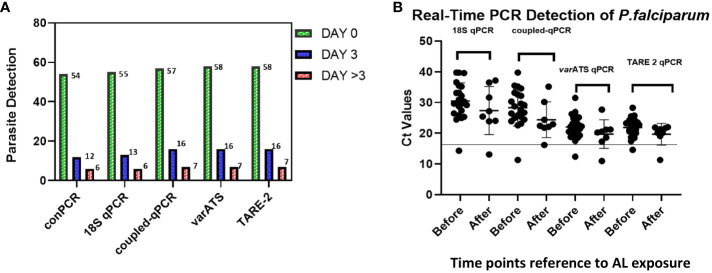
Molecular detection of *P. falciparum* in study samples before and after AL Administration **(A)** Molecular detection of *P. falciparum* infection One-way ANOVA was performed to compare the difference in parasite detection on DayO, Day3 and Day >3 across the methods. The analysis revealed that there was a statistically significant difference in mean detection between at least two timepoints (F(4, 8) = [9.385], p = 0.0001). Tukey’s HSD Test for multiple comparisons showed significant difference in detection between Day0 and Day3 (p = 0.024, 95% C.I. = [-14.48, -0.92]); between Day3 and Day >3 (p=0.0041) and between Day0 and Day>3 (p=0.0001); **(B)** Real-Time PCR confirmation of *P. falciparum* in participants samples (Pre- and Post-AL administration). Molecular detection of *P. falciparum* was done employing five different methods including the conventional 18S srRNA gene nested-PCR (conPCR) and real-time PCR (18S real-time PCR, coupled 18S conventional and real-time PCR, *var*ATS and TARE-2 real-time PCR) using gDNA from samples Pre- and Post-AL administration, with normalization with negative control ct values. A *t*-test performed for comparison of the difference in parasite detection, before and after AL administration, by real-time PCR methods showed statistically significantly higher detection before AL administration (mean=54.4, SD=3.1) than after AL administration (mean=7.2, SD=1.5), *t*(52), p=0.006.

We analyzed *P. falciparum* samples collected in a 28-day *in vivo* therapeutic efficacy studies (TES) wherein we comparatively applied 18S rRNA gene PCR, real-time PCR, *var* ATS and TARE-2 for parasite molecular detection. We investigated genetic polymorphisms on *PfCoronin* for samples showing ART tolerance. *P. falciparum* neutral microsatellite analysis here was used to confirm recrudescent infections for genetic analysis. A total of 52, out of the 58, microscopically-confirmed *P. falciparum* malaria-positive subjects were included in the *in vivo* follow-up TES. Five (8.62%) subjects did not meet the inclusion criteria while one (1.72%) was lost to follow-up at Day 7 (**Figure 2**). The median age of the participants was 5.5 years and malaria prevalence was 31.52% in the study site. A total of 7(13.46%) cases resolved as early therapeutic failure (ETF), Late Parasitological Failure (LPF) or late clinical failure (LCF) after ACT treatment (**Table 1**). These infections cleared following treatment with the rescue drug, Dihydroartemisinin-piperaquine. Two of the cases showed symptoms on Day 14, including isolate 7 (with parasitaemia on Day 14). Results from the different molecular detection assays were comparable, although the two methods targeting higher copy number genes were relatively more sensitive with lower Ct values (**Figure 3**). Conventional PCR (of Day 0 samples) showed 54 (93.1%) of the samples were positive, 55 (94.8%) by 18S Real-Time PCR, coupled-qPCR 57 (98.3%) v*ar*ATS and TARE-2 confirmed all the 58 participants’ samples positive for *P. falciparum*. Detection was significantly (ANOVA, p=0.0001) higher on Day0 than any other timepoints analyzed across the methods (**Figure 3A**); and a statistically significantly higher detection before AL administration (mean=54.4, SD=3.1) than after AL administration (mean=7.2, SD=1.5), *t*(52), p=0.006 was observed across the real-time PCR analysis methods (**Figure 3B**).

**Table 1:** Follow-up analysis.

Parameter	Value
Number of patients screened for *P. falciparum*	184
Number of patients microscopically positive for malaria	58
Prevalence of Malaria (%)	31.52
Number of participants that completed the study	52
Participants’ gender (Test) Male Female	24 (46.15%) 28 (53.85%)
Median Age (years)	5.5
< 5 years > 5 years	22 (42.31) 30 (57.68)
Positive parasitemia from day 3 (n = 52) Microscopy Day 3, first slide Day 3, second slide Day 7, first slide Day 7, second slide Day 14, first slide Day 14, second slide	(n [%]) 6 (11.54) 8 (15.38) 7 (13.46) 5 (9.62) 1 (1.92) 2 (3.85)
ETF/LCF/LPF (PCR corrected)	7 (13.46)
ACPR 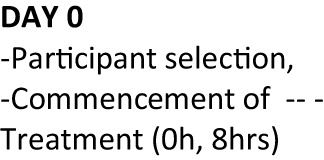 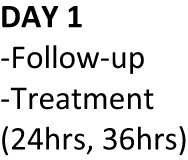 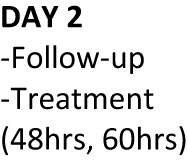 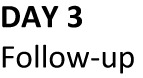 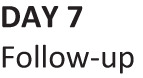 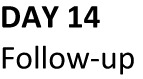	45 (86.54) 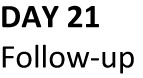 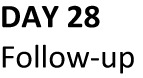

ETF, Early Treatment Failure; persistence of parasitaemia up till day 3 with axillary temperature ≥ 37.5°C; LPF, Late Parasitological Failure; parasitaemia on any day between day 7 and day 28 with axillary temperature ≥ 37.5°C; LCF, Late Clinical Failure; Late Clinical Failure (LCF) was presence of danger signs or severe malaria or parasitaemia with axillary temperature ≥37.5°C between day 4 and day 28, without having been previously classified as ETF while Adequate Clinical; ACPR, Adequate Clinical and Parasitological Response; absence of parasitaemia on day 28, in patients who did not previously meet any of the criteria for ETF or LTF.

### Genotypes of pre- and post-treatment infections

**Figure 4:**
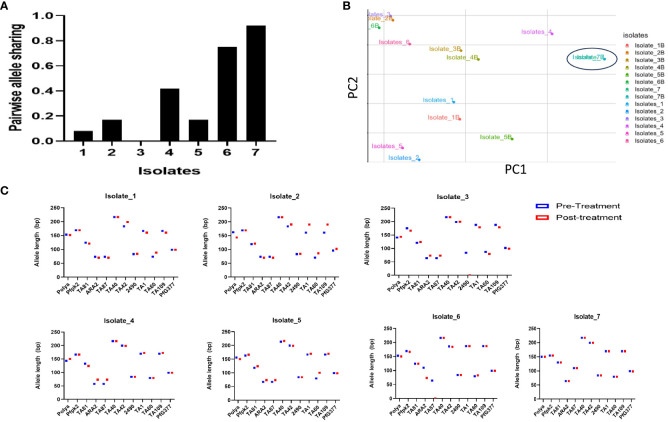
Parasite genotyping **(A)** Pairwise Allele Sharing between *P. falciparum* Pre- and Post-Treatment Infection Haplotypes; **(B)** Principal component analysis of the Isolates Pre- and Post-Treatment administration using similar *P. falciparum* neutral multilocus microsatellites; **(C)** *A*llele sizes of twelve multi-locus microsatellite markers from *P. falciparum* isolates showing ART tolerance in Nigeria. *Plasmodium falciparum* isolates were genotyped by analysis.

Results following *msp2* genotyping and agarose gel electrophoresis analysis showed three of the seven samples displaying recurrence infections during *in vivo* follow-up TES as recrudescence (Isolates 2, 5 and 7) and four re-infection (Isolates 1, 3, 4, and 6) (Additional File, (S3)). Microsatellites analysis however, confirmed only one infection as recrudescence, identical in the twelve *loci* pre- and post-AL administration (**Figures 4A–C**). Pairwise allele sharing (PAS) between the pre- and post-treatment *P. falciparum* parasites for this isolate also showed a score of 0.92 (identical in ≥ 11 loci), and another isolate a score of 0.75 (identical in nine loci) (**Figure 4A**).

Genetic diversity in terms of expected heterozygosity (*H_e_*), allelic diversity, calculated for each microsatellite locus in the isolates from the two groups (pre- and post-AL administration) revealed overall (*H_e_*): 0.8363 +/- 0.0273 and 0.584 ± 0.408 pre- and post-AL administration respectively. There was no significant difference in the overall mean allelic diversity, *He*, (P = 0.19, Mann-Whitney test) in the pre- and post-treatment groups (**Table 2**).

**Table 2:** Allelic Diversity (*He*) of *Plasmodium falciparum* isolates by microsatellite *loci*.

Locus	#Alleles		*He* (mean ± SD)	
	Pre	Post	Pre	Post
Polyα	6	3	0.52 ± 0.13	0.66 ± 0.11
Pfpk2	5	2	0.91 ± 0.24	0.52 ± 0.17
TA81	6	3	0.98 ± 0.20	0.66 ± 0.25
ARA2	6	2	0.85 ± 0.12	0.79 ± 0.18
TA87	5	2	0.90 ± 0.15	0.83 ± 0.08
TA40	3	2	0.81 ± 0.03	0.68 ± 0.19
TA42	3	4	0.85 ± 0.06	0.85 ± 0.22
2490	1	1	0.28 ± 0.11	0.4 ± 0.14
TA1	4	6	0.95 ± 0.17	0.91 ± 0.06
TA60	4	4	0.85 ± 0.07	0.93 ± 0.13
TA109	5	7	0.95 ± 0.21	0.98 ± 0.14
PfG377	3	3	0.52 ± 0.10	0.33 ± 0.03

To assess selection, LD measured as a standardized association index *I_A_^S^* between multiple *P. falciparum* loci in the study isolates revealed significant LD (*I_A_^S^* = 0.2865, *P*=0.02 and *I_A_^S^* = 0.3961, *P*=0.04 for pre- and post-treatment isolates respectively by Monte Carlo simulation method) around the neutral microsatellite loci.

### Genetic analysis of *Pfkelch-13* and coronin mutations

Sequence analysis of the *Pfcoronin* peculiar to the isolate of reduced artemisinin sensitivity, confirmed by microsatellite analysis, in this study revealed the earlier reported P76S mutation (http://www.malariagen.net) and two new (I68G and L173F) (Additional file, (S5)).

Previously reported coronin mutations (R100K/E107V) with demonstrated association with artemisinin resistance, and the Pfk13ART-resistance mutations (P574L/F446I/N458Y/Y493H/I543T/R539T/M476I/P553L/R561H/C580Y) were not detected. The *Pfcoronin* gene sequences (on Days 0,3, 7 and 14) of the isolate showing reduced sensitivity to AL in this study have been deposited in the GenBank database of National Centre for Biotechnological Information (https://www.ncbi.nlm.nih.gov/) with accession numbers MZ818700, OR344933, OR344934 and OR344935.

**Figure 5:**
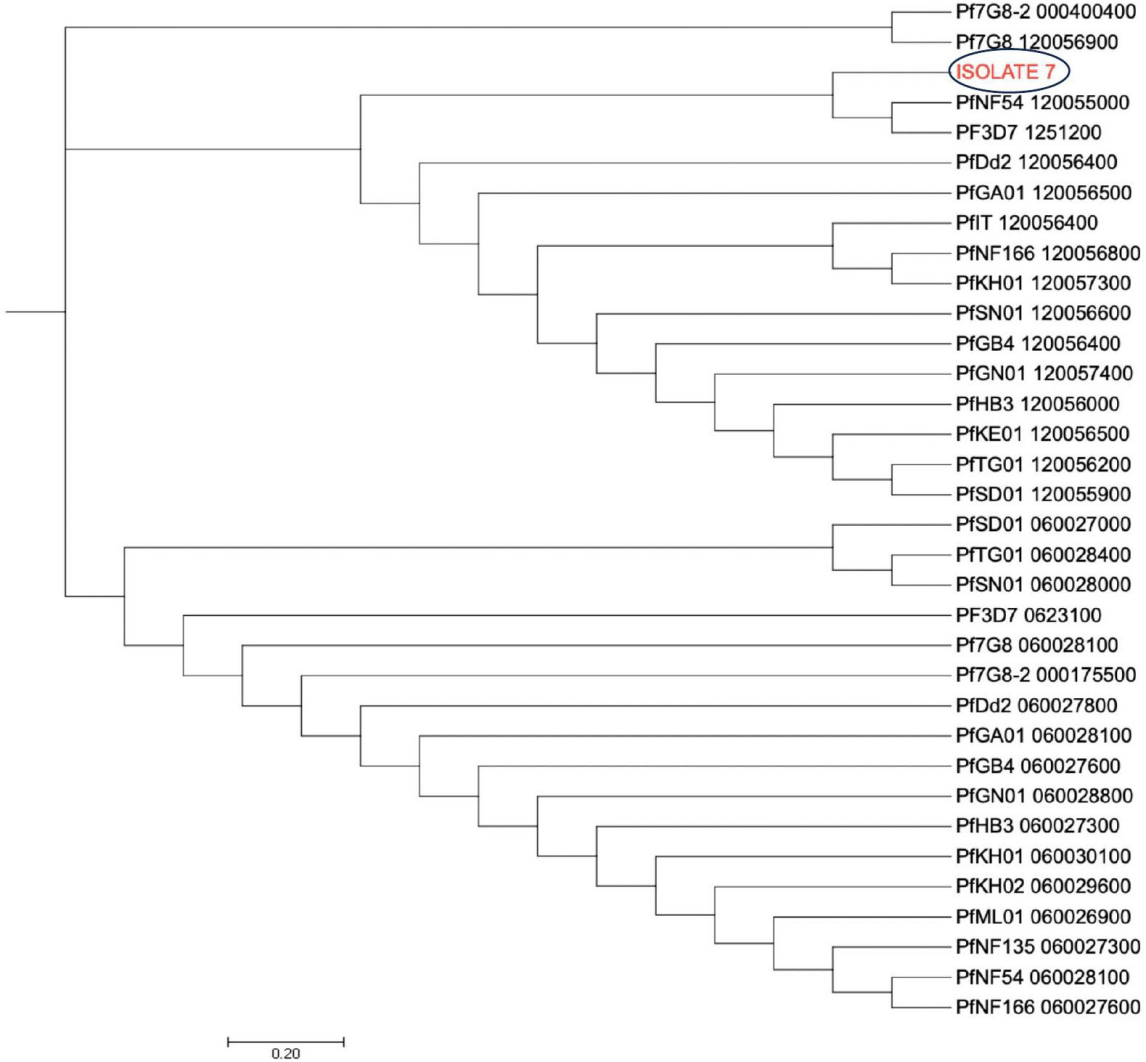
Pfcoronin genetic relatedness. The evolutionary history was inferred using the Neighbor-Joining method (26). The optimal tree with the sum of branch length = 47.00125286 is shown. The tree is drawn to scale, with branch lengths in the same units as those of the evolutionary distances used to infer the phylogenetic tree. The evolutionary distances were computed using the Maximum Composite Likelihood method and are in the units of the number of base substitutions per site. The analysis involved 6 reference nucleotide sequences and the suspected isolate sequence (Isolate 7). All positions containing gaps and missing data were eliminated. There were a total of 434 positions in the final dataset. Evolutionary analyses were conducted in MEGA7 (24).

The dendrogram with the *pfcoronin* sequences of this isolate with suspected *in vivo* artemisinin insensitivity and those of different Plasmodium isolates of *P. falciparum* strains and other species showed clustering of the isolate with Pf3D7 (PF3D7_1246200),7G8 (Pf7G8-2_000400400), PfGB4_120056400, Dd2 (PfDd2_120056400), PfHB3_120056000, *P. knowlesi* (PfKH01_120057300) and PfNF54_120055000 sequences (**Figure 5**). All other six isolates, including those earlier suspected by allelic marker genotyping as likely recrudescence, showed no variance from the wild type *Pfcoronin* allele on Days 0 and Day of parasitaemia following AL administration.

Analysis of the effects of these novel single nucleotide variants on protein stability and function of *Pfcoronin* showed that only one of the SNPs had a destabilizing effect (ΔΔG= - 0.). The remaining SNPs had positive ΔΔG values (**Table 3**).

**Table 3:** Protein stability.

S/N	Variant (SNPs)	*ΔΔG (kcal mol-1)	Location	Status
3	I68G	1.84	COR	New
5	P76S	0.86	SUR	Reported
6	L173F	-0.48	COR	New

COR, Core region; SUR, Surface region.

*ΔΔG, Delta delta G.

### Drug resistance typing

HRM typing of drug resistance associated markers for pre- and post-treatment parasite genotyping showed that almost all the samples had the drug-resistance haplotypes 108**_N/T_** (92.86%), 59**_R_** (100%) and 51**_I_** (%) in the *Pfdhfr* codons, however, only two isolates carried the drug resistance mutant allele I164L (14.29%). The *Pfdhps* 613T and 581G mutations were present in 50% and 7.14% of the parasite isolates, respectively. *Pfmdr1* 86Y and 184F mutations associated with reduced parasite sensitivity to lumefantrine were observed in 71.42% and 65.29% respectively, in the isolates.

Two isolates were observed to exhibit a quintuple *Pfdhfr/Pfdhps* haplotypes (108**_T/N_**/51**_I_**/164**_L_**/59**_R_**/581**_G_**) in addition to *Pfmdr1* double haplotype (86**_Y_**/184**_F_**) (**Table 4**). One of the isolates with this combined septuple haplotype showed parasitaemia post treatment and genotype features depicting recrudescence. All samples had the *Pfcrt* 76T drug resistance allele.

**Table 4:** Antimalarial drug resistance target typing of pre- and post-treatment isolates.

Sample ID	Outcome	DHFR CODON				DHPS CODON		MDR1 CODON		K13 CODON	CRT CODONS	CORONIN CODONS
	(*WHO 2003)	N51I	C59R	S108N/T	I164L	A581G	A613S/T	N86Y	Y184F	N458Y, Y493H, I543T, R539T, M476I, P553L, R561H, C580Y	K76T	G50E/R100K/E107V
**S1**	ETF	**I**	**R**	**N**	**I**	**A**	**S**	N	F	WT	MUT	WT
**S2**	LPF	**I**	**R**	**N**	**I**	**A**	**A**	**Y**	**F**	WT	MUT	WT
**S3**	ETF	**I**	**R**	**T**	**I**	**A**	**A**	**Y**	**Y**	WT	MUT	WT
**S4**	LPF	**I**	**R**	**T**	**I**	**A**	**A**	**N**	**F**	WT	MUT	WT
**S5**	ETF	**I**	**R**	**N**	**I**	**A**	**A+T**	**Y**	**F**	WT	MUT	WT
**S6**	LPF	**I**	**R**	**T**	**I**	**A**	**A**	**Y**	**F**	WT	MUT	WT
S7*	LPF	**I**	**R**	**N**	**L**	**A**	**S**	**Y**	**Y**	WT	MUT	WT/MUT
**S1B**	ETF	**I**	**R**	**T**	**I**	**A**	**A**	**Y**	**F**	WT	MUT	WT
**S2B**	LCF	**I**	**R**	**T**	**I**	**A**	**A**	**Y**	**F**	WT	MUT	WT
**S3B**	ETF	**I**	**R**	**N**	**I**	**G**	**S**	**N**	**F**	WT	MUT	WT
**S4B**	ETF	**I**	**R**	**N**	**I**	**A**	**A+T**	**Y**	**F**	WT	MUT	WT
**S5B**	LCF	**I**	**R**	**N**	**I**	**A**	**T**	**Unknown**	**Unknown**	WT	MUT	WT
**S6B**	LPF	**I**	**R**	**Unknown**	**I**	**A**	**T**	**Y**	**Unknown**	WT	MUT	WT
S7B*	LPF	**I**	**R**	**N**	**L**	**A**	**S**	**Y**	**Y**	WT	MUT	WT/MUT
**MUT (%)**		100	100	92.86	14.29	7.14	50	71.42	65.29	0	100	0

*: Sample showing recrudescence and evidence of resistance phenotype; K13 mutations: 441-726; WT, wild type; MUT, mutant allele.

S1,S2,S3,S4,S5,S6: pre- drug administration.

S1B, S2B, S3B, S4B,S5B,S6B.S7B: Post- drug administration.

## Discussion

Antimalarial drug resistance threatens to continue eroding the traction gained by global malaria control and elimination efforts over the last decade. Its spread and reduced efficacy to the current ACTs will have a significant global health impact with potentially more devastating effect in Nigeria and other African countries with a disproportionately high burden of malaria morbidity and mortality.

This study, conducted in Lagos, South-west Nigeria, characterized *P. falciparum* isolates with suspected tolerance to Artermether-Lumefantrine following a 28-day *in vivo* TES. The goal was to investigate the role of parasite molecular mediators of reduced *in vivo* ART efficacy of AL against *P. falciparum* parasites in Nigeria. After a 3-day AL administration and 28-day follow-up, we observed *P. falciparum* recrudescence in a single clinical isolate among seven different subjects showing post treatment parasitaemia. Three cases of therapeutic failure of AL were reported before in Benin, Nigeria, a South-South city located about 321 km from Lagos, the site of the current study (Ebohon et al., 2019). Lagos is cosmopolitan, with significant in-ward urban migration that may bring in such parasites. This report and others in Africa call for more vigilance against ACT resistance in Africa, where *Pfkelch-13* and other non-Kelch mediators of artemisinin tolerance are increasingly being reported (Normile, 2021).

Parasite DNA was detected in 27% of cases post-ACT administration. These could be residual DNA, or persistent asexual stages, that could be indicative of treatment tolerance and therefore should be further investigated as early indication of suspected ART-tolerant strains of *P. falciparum* populations from Nigeria. This is consistent with the report by Roth et al. (2018), using qPCR approach to monitor persistence of parasites in patients’ blood after drug administration. In this present study, recrudescent parasitaemia was confirmed only in a single infection, the implication of this is that, given the relatively small total number of participants followed up, parasites potential to tolerate ACT in Lagos and metropolis may be on the increase. In this setting, self-medication is prevalent, sub-optimal blood concentrations of these antimalarial drugs usually result in drug-tolerant, mostly asymptomatic, submicroscopic parasitaemia.

Molecular diagnostic techniques are more sensitive in detecting low levels of parasitemia. Though they are cumbersome, their sensitivity and cost-effectiveness, and deployment to detect viable parasites allows for application in monitoring antimalarial drug treatment. All four techniques used in this study showed reduction in post-treatment parasite DNA in blood samples, with the most sensitive being TARE-2 qPCR. Previous reports had shown TARE-2 to be less sensitive than other methods such as *var*-ATS in detecting low levels of parasitemia (Haanshuus et al., 2019). Moreover, TARE-2, a SYBR green based real-time PCR method, is less costly, in comparison with the Taqman *var*-ATS method. Analysis in the current study revealed that TARE-2, a multi-copy parasite gene, is equally as sensitive as the ultrasensitive *var-ATS* diagnostic marker. The application of this method in field settings may therefore need further investigation to maximize the benefits of its sensitivity and cost effectiveness in resource limited settings.

Further analysis based on twelve *P. falciparum* neutral microsatellite *loci*, revealed only one of the seven suspected post treatment infections could be classified as recrudescence. The remaining six were new or superinfection parasite isolates appearing in the peripheral blood within 28 days of ACT use. This implies that, although, *P. falciparum* parasites with reduced ART sensitivity may currently be few in Nigeria, they exist, and emerging.

Unexpectedly, *msp2* genotypes did not show multi-clonality in the samples. Though Nigeria is a high transmission setting, where multiclonality is expected, the cases here were recruited in a semi urban setting, where transmission is relatively lower and could be the reason for the low complexity of infections. *P. falciparum* deploys several mechanisms to evade drugs, including dormancy against artemisinin derivatives, where a small sub-population of asexual parasites stop growing and dividing for days or weeks following drug exposure (Maiga et al., 2021). In general, ART resistance reflects an increased propensity for dormancy, but it is not clear whether very high efficacy of ACT outside areas of ART resistance suggests that dormant forms (or more likely these parasites when they wake) do not survive the residual concentrations of partner drugs (AL). This could be the case in Africa, where despite use of ACTs for over a decade, resistance to ART is not yet common. In this study, about 86% of cases with persistent parasite DNA (the suspected ACT tolerant isolates) could be classified as reinfection and this was within the half-life of Lumefantrine, the partner drug with Artemether in AL. Hence, they could in addition be tolerant to Lumefantrine.

Tolerance to ART partner drugs such as Mefloquine, Piperaquine and Lumefantrine are well known. Except in the case of Piperaquine in South East Asia, this has not been associated with clinical failure of ACTs (van der Pluijm et al., 2019; Maiga et al., 2021). Tolerance to Lumefantrine in Africa will affect the efficacy of AL, the most common ACT in Nigeria and Africa. It is also known that Lumefantrine selects for wild type variants of markers in *Pfcrt* (K76T) and *Pfmdr1* (N86Y) that are associated with Chloroquine resistance (Bhagwat and Aravind, 2007; Adamu et al., 2020). The only isolates in suspected failure to AL carried the mutant variants at these markers. Hence, the potential failure in response to treatment may not be related to Lumefantrine tolerance but rather to patient factors or the most unlikely insensitivity to artemether.

Several other known drug resistance-associated mutations, *pfdhfr/pfdhps/pfmdr1* (108**_T/N/_** 51**_I/_** 164**_L/_** 59**_R/_** 581**_G/_** 86**_Y_**/184**_F_**) (Daniels et al., 2012), were observed in the isolates with persistent parasite DNA. The drug resistance-associated haplotype *pfdhfr/pfdhps/pfmdr1* affects the efficacy of several antimalarial drugs. As most of the isolates carried the mutant genotypes, this could have facilitated re-infection within the 28 days post AL administration.

The study found significant LD (nonrandom association between loci) around the neutral microsatellite loci, This finding is an indication that specific alleles or haplotypes of relevant genes are prevalent in this population, and might have expanded in the population. This implies a fitness advantage under the present antimalarial drug pressures. This may portend undesirable consequences on ART tolerance. Although, none of the validated mutations associated with ART resistance, with respect to *pfk13*, was detected, monitoring for these and mutations in other markers, especially Pfcoronin, is important to mitigate against resistance.

No *Pfkelch-13* ART resistance-associated variants were found in any of the persistent parasitemia isolates from this study. However, three non-synonymous Pfcoronin mutations were observed in the single infection suspected to be recrudescence and tolerant to AL. Only one of the variants had been described before, while two others were novel. *Pfcoronin* is an actin binding protein and three mutant forms selected on the background of Senegalese isolates show increased survival against dihydroartemisinin (DHA) in *in vitro* selection and CRISPR-Cas9 genetic editing experiments (Demas et al., 2018).

Though the variants found here differ, they are within the WD domain and therefore could alter the function of the protein and increase ART tolerance. The implication of this is predicated on the potential of Nigerian *P. falciparum* population for high gene flow rate, and circulation of resistance-associated SNVs. From structural models of the mutant forms of the protein, one of the variants (L173F) had a significant effect on the protein structure. More molecular and therapeutic response analysis from this population, as well as host and parasite factors underlying reduced ACT efficacy are urgently needed in the site studied here and across Nigeria. This is important as malaria is highly prevalent in Nigeria, and access to drugs in the informal sector is easy with resultant negative effects on sustained drug efficacy.

ACTs are the only WHO approved effective first-line drug against uncomplicated malaria. However, resistance to ART may be emerging or spreading as in the case of chloroquine (Haanshuus et al., 2019; Maiga et al., 2021). Although the WHO had encouraged all endemic countries to actively conduct ACT resistance surveillance, the will, resources and capacity to actualize this may be non-existent in most endemic African countries. The situation is currently worsened by the COVID-19 pandemic, with potential unclear impact on treatment outcomes and antimalarial drug metabolism and efficacy. A major aim of the global technical strategy (GTS) for malaria for a reduction in malaria case incidence and mortality rate of 75% by 2025 and 90% by 2030, from the 2015 baseline (WHO, 2023), may therefore be defeated.

Two non-synonymous nucleotide variants, with resultant codon mutations, were observed in the isolate implicated in the recrudescence infection in this study. Apart from the P76S variant, the. other two are new variants. Structure-based protein stability analysis conducted in relation to function, revealed a general stabilizing effect (Chen et al., 2020), but the L173F SNV had a destabilizing influence on the actin-binding ability of *Pfcoronin*. Whether this has an overall relationship with the parasite’s sensitivity to drug or not is currently unclear.

Although, Pfcoronin has structural similarity with Pfkelch-13, it is functionally distinct. Its biological function outside of F-actin organization remains unconfirmed. *In vitro*, *pfcoronin* gene mutations *knockin* has shown the effects of polymorphisms on the functional role of Pfcoronin in parasite ART tolerance. Pfcoronin implication in antimalarial drug resistance implies a catastrophic consequence on malaria eradication globally. This will be more debilitating in Nigeria where 27% of the global malaria burden resides.

Phylogenetic analysis of the AL-insensitive isolate from this study showed clustering and evolutionary relationship with known strains of *P. falciparum* including the Brazilian 7G8 strain. Studies focused on parasite genomic mediators of *in vivo* ART tolerance have only been minimally carried out or reported in Nigeria. To conclude, this current study confirmed that Nigerian *P. falciparum* populations are unique and complex, and reported novel SNPs in Pfcoronin as a possible kelch13-independent ART-resistance mechanism. Findings from this study would inform national policy on health research for attention focused on both known and novel ACT resistance factors. These findings suggest to relevant agencies in the country, and other endemic countries, to put in place necessary intervention and control measures to stop the spread of artemisinin or ACT tolerance among populations in the country to fast track malaria elimination.

Further studies with larger sample size are required to elucidate complex parasite factors driving reduced sensitivity to ART in Nigeria. Although efforts are ongoing to explore novel targets for discovery of new antimalarials (Komatsuya et al., 2013; Suzuki et al., 2015; Hartuti et al., 2018), the present report corroborates the need for routine surveillance for drug resistance signatures and patterns in endemic regions.

## Data availability statement

The datasets presented in this study can be found in online repositories. The names of the repository/repositories and accession number(s) can be found in the article/**Supplementary Material**.

## Ethics statement

The studies involving humans were approved by The Nigerian Institute of Medical Research Institutional Ethics review Board. The studies were conducted in accordance with the local legislation and institutional requirements. Written informed consent for participation in this study was provided by the participants’ legal guardians/next of kin. Written informed consent was obtained from the individual(s), and minor(s)’ legal guardian/next of kin, for the publication of any potentially identifiable images or data included in this article.

## Author contributions

OAj: Conceptualization, Data curation, Formal analysis, Funding acquisition, Investigation, Methodology, Project administration, Resources, Validation, Writing – original draft, Writing – review & editing. YO: Investigation, Supervision, Writing – review & editing. EO: Data curation, Formal analysis, Investigation, Writing – review & editing. MO: Data curation, Writing – review & editing. BI: Supervision, Writing – review & editing. IN: Formal analysis, Investigation, Methodology, Writing – review & editing. CN: Formal analysis, Investigation, Writing – review & editing. OA: Data curation, Investigation, Project administration, Writing – review & editing. SC: Writing – review & editing. JO: Writing – review & editing. EG: Formal analysis, Writing – review & editing. EB: Investigation, Supervision, Writing – review & editing. AA-N: Funding acquisition, Investigation, Methodology, Project administration, Supervision, Writing – original draft, Writing – review & editing.
