# Supplementary material for: Detection of novel Plasmodium falciparum coronin gene mutations in a recrudescent ACT-treated patient in South-Western Nigeria
Source: Front Cell Infect Microbiol. 2024 Apr 23;14:1366563. doi: 10.3389/fcimb.2024.1366563 (PMC11074373; doi:10.3389/fcimb.2024.1366563)
Supplement: Supplementary file 1 [file DataSheet_1.doc]

**Additional Files**

**S 1: Primers for Microsatellite hemi-nested PCR**

|  |  |  | **Fluorescent** |
| --- | --- | --- | --- |
| **Multiplex** | **Locus** | **primer 5' to 3'** | **Label** |
|  | Poly α-R | ATCAGATAATTGTTGGTA |  |
| **1** | Poly α-F | AAAATATAGACGAACAGA | FAM |
|  | Poly α-3(IR) | GAAATTATAACTCTACCA |  |
|  | PFPK2-3R | CCTCAGACTGAAATGCAT |  |
| **1** | PFPK2-F | CTTTCATCGATACTACGA | HEX |
|  | PFPK2-R | AAAGAAGGAACAAGCAGA |  |
|  | TA81-3F | GAAGAAATAAGGGAAGGT |  |
| **1** | TA81-R | TTTCACACAACACAGGATT | PET |
|  | TAA81-F | TGGACAAATGGGAAAGGATA |  |
|  | ARA2-3(F) | GTACATATGAATCACCAA |  |
| **2** | ARA2-R | GCTTTGAGTATTATTAATA | FAM |
|  | ARA2-F | GAATAAACAAAGTATTGCT |  |
|  | TA87-3F | ATGGGTTAAATGAGGTACA |  |
| **2** | TA87-R | ACATGTTCATATTACTCAC | HEX |
|  | TA87-F | AATGGCAACACCATTCAAC |  |
|  | TA40 Rev-1 | GAAATTGGCACCACCACA |  |
| **2** | TA40 For | AAGGGATTGCTGCAAGGT | PET |
|  | TA40 Rev-2 | CATCAATAAAATCACTACTA |  |
|  | TA42-3F | ACAAAAGGGTGGTGATTCT |  |
| **3** | TA42-R | GTATTATTACTACTACTAAAG | FAM |
|  | TA42-F | TAGAAACAGGAATGATACG |  |
|  | 2490-3R | ATGATGTGCAGATGACGA |  |
| **3** | 2490-F | TTCTAAATAGATCCAAAG | HEX |
|  | 2490-R | TAGAATTATTGAATGCAC |  |
|  | TA1-3(F) | CTACATGCCTAATGAGCA |  |
| **3** | TA1-R | TTTTATCTTCATCCCCAC | PET |
|  | TA1-F | CCGTCATAAGTGCAGAGC |  |
|  | TA60-F | CTCAAAGAAAAATAATTCA |  |
| **4** | TA60-R | AAAAAGGAGGATAAATACAT | FAM |
|  | TA60-3(IF) | TAGTAACGATGTTGACAA |  |
|  | TA109-3F | TAGGGAACATCATAAGGAT |  |
| **4** | TA109-R | CCTATACCAAACATGCTAAA | HEX |
|  | TA109-F | GGTTAAATCAGGACAACAT |  |
|  | PFG377-3R | TTATGTTGGTACCGTGTA |  |
| **4** | PFG377-F | GATCTCAACGGAAATTAT | PET |
|  | PFG377-R | TTATCCCTACGATTAACA |  |

**S2: Table 1:** Oligonucleotide primers for drug Resistance Typing.

| **Gene** | **Assay** | **Forward primer (5′→3′)** | **Reverse primer (5′→3′)** |
| --- | --- | --- | --- |
|  | **SNP ID** |  |  |
| *PfDHFR* | N51/C59 | ACATTTAGAGGTCTAGGAAATAAA | ATATTTACATCTCTTATATTTCAATTTTTCA |
|  |  | GGAGT | TATTTTGATTCATTCAC |
|  | I164 | ACAAAGTTGAAGATCTAATAGTTT | CTGGAAAAAATACATCACATTCATATGTAC |
|  |  | TACTTGGG | TATTTATTCTA |
|  | S108 | CTGTGGATAATGTAAATGATATGC | GACAATATAACATTTATCCTATTGCTTAAA |
| *PfDHPS* |  | CTAATTCTA | GGT |
|  | K540 | GTGTTGATAATGATTTAGTTGATA | GTTTATCCATTGTATGTGGATTTCCTCTT |
|  |  | TATTAAATGATATTAGTGC |  |
|  | A581 | CTTGTATTAAATGGAATACCTCGT | AGTGGATACTCATCATATACATGTATATTT |
|  |  | TATAGGA | TGTAAG |
| *PfMDR* | N86 | TTATTATTTATATCATTTGTATGTG | CAGGAAACAGCTATGACATCATTGATAATA |
|  |  | CTGTATTATCAGG | TAAATTGTACTAAACCTATAGATACT |
|  | Y184 | AGTTCAGGAATTGGTACGAAATTT | ACGCAAGTAATACATAAAGTCAAACG |
|  |  | ATAACA |  |

**S3**

**
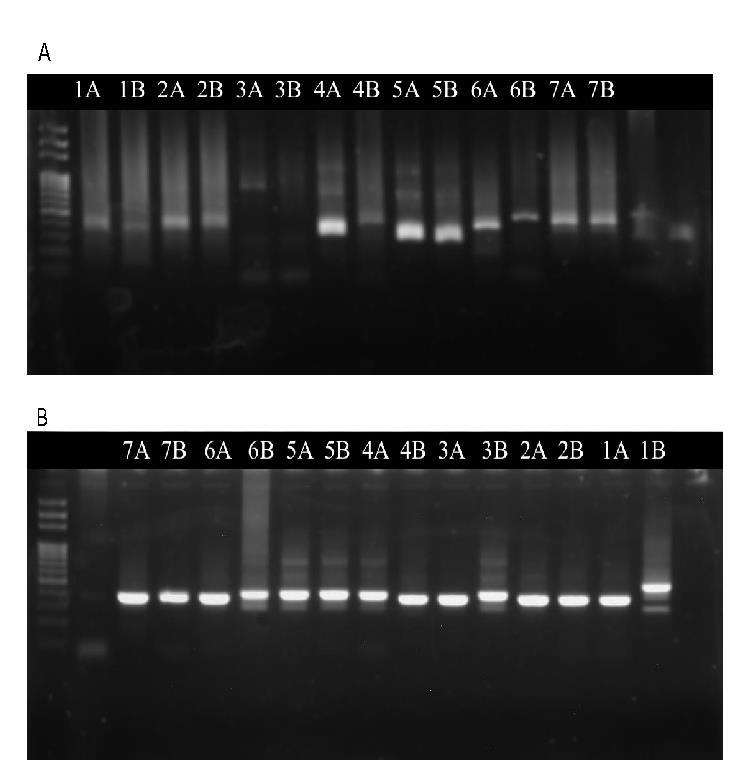
**

**Plate 1: Electropherogram of *P. falciparum msp2* nested-PCR amplicons resolved on 1% agarose gel.** (A) 3D7 family amplification: Three samples (2, 5 and 7) showed recrudescence infections with similar Day 0 (‘A’ or Pre - AL administration) and Day 3+ (‘B’ or Post-AL administration) parasite isolates. (B) FC27 family amplification: Three samples (2, 5 and 7) showed recrudescence infections with similar Day 0 (‘A’ or Pre - AL administration) and Day 3+ (‘B’ or Post-AL administration) parasite isolates.

**S4**

|  |  | **REF** | **Alternative** | **Amino Acid** | **Nigeria** |
| --- | --- | --- | --- | --- | --- |
| **S/N** | **Position** | **(3D7)** |  | **change** | Field |
|  |  |  |  |  | Isolate |
|  |  |  |  |  |  |
| 1 | 2092279 | C | G | N17K | N |
| 2 | 2092606 | G | A | V62M | V |
| 3 | 2092624 | T | G, A | I68G | G |
| 4 | 2092648 | C | T, G | P76S | S |
| 5 | 2092751 | A | C | N110T | N |
| 6 | 2092768 | G | A | D116N | D |
| 7 | 2092938 | A | C | L173F | F |
| 8 | 2092969 | A | G | S183G | S |
| 9 | 2093131 | A | C | N237H | N |
| 10 | 2093209 | G | T | A263S | A |

**Table S4: Pfcoronin SNPS in Comparison with earlier reported SNPs from MalariaGEN.**

Position: Genomic location; REF: *Plasmodium falciparum* 3D7 reference sequence amino acid; Alternative=Variants other than on 3D7 sequence; Nigeria Field Isolate: amino acid observed on Nigerian parasite isolates

**Figure S5: Pfcoronin SNVs from the Study Site**
